# Thyroid hormones and modulation of diastolic function: a promising target for heart failure with preserved ejection fraction

**DOI:** 10.1177/2042018820958331

**Published:** 2020-10-04

**Authors:** João Sérgio Neves, Catarina Vale, Madalena von Hafe, Marta Borges-Canha, Ana Rita Leite, João Almeida-Coelho, André Lourenço, Inês Falcão-Pires, Davide Carvalho, Adelino Leite-Moreira

**Affiliations:** Departamento de Cirurgia e Fisiologia, Unidade de Investigação Cardiovascular, Faculdade de Medicina, Universidade do Porto, Porto, Portugal; Department of Endocrinology, Diabetes and Metabolism, Centro Hospitalar Universitário de São João, Faculdade de Medicina, Universidade do Porto, Porto, Portugal; Departamento de Cirurgia e Fisiologia, Unidade de Investigação Cardiovascular, Faculdade de Medicina, Universidade do Porto, Porto, Portugal; Departamento de Cirurgia e Fisiologia, Unidade de Investigação Cardiovascular, Faculdade de Medicina, Universidade do Porto, Porto, Portugal; Departamento de Cirurgia e Fisiologia, Unidade de Investigação Cardiovascular, Faculdade de Medicina, Universidade do Porto, Porto, Portugal; Department of Endocrinology, Diabetes and Metabolism, Centro Hospitalar Universitário de São João, Faculdade de Medicina, Universidade do Porto, Porto, Portugal; Departamento de Cirurgia e Fisiologia, Unidade de Investigação Cardiovascular, Faculdade de Medicina, Universidade do Porto, Porto, Portugal; Departamento de Cirurgia e Fisiologia, Unidade de Investigação Cardiovascular, Faculdade de Medicina, Universidade do Porto, Porto, Portugal; Departamento de Cirurgia e Fisiologia, Unidade de Investigação Cardiovascular, Faculdade de Medicina, Universidade do Porto, Porto, Portugal; Departamento de Cirurgia e Fisiologia, Unidade de Investigação Cardiovascular, Faculdade de Medicina, Universidade do Porto, Porto, Portugal; Department of Endocrinology, Diabetes and Metabolism, Centro Hospitalar Universitário de São João, Faculdade de Medicina, Universidade do Porto, Porto, Portugal; Instituto de Investigação e Inovação em Saúde da Universidade do Porto, Portugal; Departamento de Cirurgia e Fisiologia, Unidade de Investigação Cardiovascular, Faculdade de Medicina, Universidade do Porto, Alameda Hernâni Monteiro, Porto, 4200-319, Portugal

**Keywords:** diastolic function, heart failure, hypothyroidism, non-thyroidal illness syndrome, thyroid hormones

## Abstract

Heart failure with preserved ejection fraction (HFpEF) is a clinical syndrome with high mortality for which there is no proven therapy to improve its prognosis. Thyroid dysfunction is common in heart failure (HF) and is associated with worse prognosis. In this review, we discuss the cardiovascular effects of thyroid hormones, the pathophysiology of HFpEF, the prognostic impact of thyroid function, and the potential of thyroid hormones for treatment of HFpEF. Thyroid hormones have a central role in cardiovascular homeostasis, improving cardiac function through genomic and non-genomic mechanisms. Both overt and subclinical hypothyroidism are associated with increased risk of HF. Even when plasmatic thyroid hormones levels are normal, patients with HF may have local cardiac hypothyroidism due to upregulation of type 3 iodothyronine deiodinase. Thyroid hormones improve several pathophysiological mechanisms of HFpEF, including diastolic dysfunction and extra-cardiac abnormalities. Supplementation with thyroid hormones (levothyroxine and/or liothyronine), modulation of deiodinase activity, and heart-specific thyroid receptor agonists are potential therapeutic approaches for the treatment of HFpEF. Further preclinical and clinical studies are needed to clarify the role of thyroid hormones in the treatment of HFpEF.

## Introduction

Thyroid hormones have a central role in cardiovascular system development and homeostasis. Both hypothyroidism and hyperthyroidism are associated with characteristic cardiovascular changes and even subclinical dysfunction is known to increase cardiovascular risk.^1^

Heart failure (HF) is the final stage of several cardiovascular conditions, affecting over 23 million people worldwide.^2^ HF can be divided into two major entities according to the ejection fraction (EF): HF with reduced EF (HFrEF) and HF with preserved EF (HFpEF). The latter is responsible for over 50% of all cases. Like HFrEF, HFpEF is associated with decreased functional capacity, decreased quality of life, and high mortality. However, the pathophysiology of HFpEF is less well understood and there is as yet no proven therapy to improve its prognosis. Although its core feature was long held to be diastolic dysfunction, systemic disturbances that jeopardize cardiovascular reserve may also constitute essential pathophysiological mechanisms.^3^

In this review, we discuss the cardiovascular effects of thyroid hormones, the pathophysiology of HFpEF, the prognostic impact of thyroid function, and the potential of thyroid hormones for treatment of HFpEF.

## Cardiovascular effects of thyroid hormones

Thyroid hormones modulate the cardiovascular system by genomic and non-genomic mechanisms.^4^ The thyroid gland produces thyroxine (T4) hormone in greater quantity than triiodothyronine (T3), at a ratio of 10:1. T3 is biologically more active than T4 and is considered the active form of thyroid hormones.^5^ The primary mechanism of action of T3 is the interaction with thyroid hormone receptors (TR) – a process that can either enhance or repress the transcription of specific target genes.^4^ There are two TR genes (TRα and TRβ) with specific patterns of expression in different tissues. Both genes produce different isoforms as a result of alternative splicing.^6^ TRα1 is expressed predominantly in brain and heart. TRβ1 is expressed in liver, kidney, and skeletal muscles, and, at lower levels, in most tissues including the heart. On the other hand, TRβ2 is expressed predominantly in brain, pituitary gland, retina, and inner ear, and appears to be important for regulating the negative-feedback loop of the hypothalamus-pituitary-thyroid axis.^4,6^ About 80% of the circulating T3 is produced in peripheral tissues by conversion of T4. This conversion is mediated by tissue deiodinases. Type 1 and type 2 deiodinases (D1 and 2, respectively) mainly convert T4 into T3, while type 3 deiodinase (D3) converts T4 and T3 into the functionally inactive reverse T3 (rT3) and 3,3-diiodothyronine (T2), respectively.^7,8^ D3 has higher affinity in inactivating T3 and plays a critical role in regulating T3 availability.^9^ Deiodinases regulate both serum and intracellular tissue levels of thyroid hormones. Several conditions, including chronic inflammation, neoplastic diseases, chronic kidney disease, myocardial ischemia, and HF, alter the pattern of deiodinase activity, increasing the conversion of T4 into rT3 and decreasing the availability of T3.^8,10,11–13^ T3 improves systolic and diastolic myocardial function and increases heart rate. Thyroid hormones enhance the expression of genes encoding sarco/endoplasmic reticulum calcium-ATPase (SERCA2a), fast α-isoform of myosin heavy chain (α-MHC), Na+/K+ ATPase, and voltage-gated K+ channels (Kv1.5 and Kv4.2), and negatively regulates the transcription of phospholamban (PLN) and slow β-isoformof myosin heavy chain (β-MHC).^14^ Both myosin heavy chains are components of the cardiac contractile apparatus, and this change in expression pattern results in an increased velocity of contraction.^15^ The increase of SERCA2a and the inhibition of PLN increase the calcium available for systolic contraction, and improve the reuptake of calcium into the sarcoplasmic reticulum during relaxation of the heart.^15^ Efficient kinetics of calcium is indispensable for energetically optimal cardiac myocyte relaxation and contraction. Furthermore, thyroid hormones increase the gene expression of the β-adrenergic receptors, enhancing the response to catecholamines, which act in synergy with thyroid hormones.^16^ Thyroid hormones also protect the heart from ischemic lesion by decreasing coronary resistance, reducing the activation of the pro-apoptotic p38 MAPK signaling pathway and increasing the activity of myocardial PKCδ and the expression of heat shock proteins 27 and 70.^17^ In addition, thyroid hormones stimulate cell growth and neo-angiogenesis, and decrease cardiac fibrosis by enhancing metalloproteinase and antifibrotic effects.^17^

The effects of thyroid hormones on the vasculature include genomic and non-genomic mechanisms. Non-genomic effects include ion channel modulation and regulation of specific transduction pathways. In vessels, thyroid hormones activate phosphatidylinositol 3-kinase (PI3K)/serine/threonine-protein kinase (AKT) signaling pathways enhancing nitric oxide production by endothelial cells and activate non-genomic pathways that induce smooth muscle relaxation, thereby decreasing vascular resistance and left ventricular (LV) afterload.^18^ The decrease in systemic vascular resistance, coupled with the inotropic effects, leads to an increase in cardiac output.^19^

Thyroid hormones also have favorable effects on plasma lipid profile, which may decrease the risk of atherosclerosis development and progression.^1^ This beneficial effect on the lipid profile is due to the increase of sterol regulatory element-binding protein-2 (SREBP-2), which regulates the expression of the LDL receptors.^20^

## Cardiovascular manifestations in thyroid dysfunction

Given the known effects of thyroid hormones on the cardiovascular system, the association of thyroid dysfunction with cardiovascular changes has been evaluated by many studies. These associations are better established in overt thyroid dysfunction than in subclinical dysfunction. Table 1 summarizes the cardiovascular changes in thyroid dysfunction.

**Table 1.** Cardiovascular changes, comorbidities and mortality in thyroid dysfunction.

	Overt hypothyroidism	Subclinical hypothyroidism	Subclinical hyperthyroidism	Overt hyperthyroidism
Systolic dysfunction	↑↑	↑	↓/↑	↓/↑
Diastolic dysfunction	↑↑	↑↑	↓/↑	↓/↑
Heart rate	↓↓	↓	↑	↑↑
Hypertension	↑ (diastolic)	↑ (diastolic)	↑ (systolic)	↑ (systolic)
Dyslipidemia	↑↑	↑	↓	↓
Heart failure	↑↑	↑	–/↑	↑↑
Coronary artery disease	↑↑	↑	–/↑	–/↑
Atrial fibrillation	–/↓	–/↓	↑	↑↑
Atherosclerosis	↑↑	↑	–/↑	–/↑
Pulmonary hypertension	–	–	–	↑
Cardiovascular mortality	↑	–/↑	–/↑	↑
All-cause mortality	↑	–/↑	–/↑	↑

↑↑: markedly increased; ↑: increased; –/↑: possibly increased; –: no effect; –/↓: possibly decreased; ↓/↑: possibly decreased or increased; ↓: decreased; ↓↓: markedly decreased. See text for details.

Subclinical hypothyroidism is defined as elevated TSH with normal levels of free T4. The results of studies evaluating the effects of subclinical hypothyroidism on the cardiovascular system are inconsistent. Some, but not all, have shown increased all-cause and cardiovascular mortality, higher risk of coronary heart disease and HF.^21–24^ Most studies suggest that the risk of adverse cardiovascular outcomes is higher when TSH ⩾ 10 mlU/l.^22^ In the Penn Heart Failure Study, a prospective cohort of patients with HFrEF and HFpEF, TSH ⩾ 7 mlU/l was associated with an increased risk of a composite end point of ventricular assist device placement, heart transplantation, or death in patients.^25^ Subclinical hypothyroidism has been associated with impaired systolic and diastolic cardiac function, increased carotid artery intima-media thickness, vascular dysfunction, and higher blood pressure.^26–28^ On the contrary, subclinical hypothyroidism may be associated with a lower risk of atrial fibrillation.^29^

Overt hypothyroidism is defined as high TSH with low free T4.^1^ In most studies, it has been associated with increased risk of HF, coronary artery disease, and all-cause and cardiovascular mortality.^30,31^ Overt hypothyroidism is associated with decreased cardiac output and contractility, lower heart rate, and higher systemic vascular resistance.^30^ Diastolic dysfunction is a characteristic feature in most studies.^32,33^ Cardiovascular risk factors are amplified in patients with overt hypothyroidism, particularly diastolic hypertension and dyslipidemia. Most studies have also shown increased carotid artery intima-media thickness in overt hypothyroidism.^34,35^

Subclinical hyperthyroidism is defined by low TSH with normal free T4.^1^ It has been associated with a higher risk of cardiovascular disease, including coronary events, HF, and atrial fibrillation.^36,37^ Some studies showed an increased risk of all-cause and cardiovascular mortality in patients with subclinical hyperthyroidism, but others have shown no association.^36,38–40^ The strongest association of subclinical hyperthyroidism appears to be with atrial fibrillation. However, some studies suggest that this association may only be seen when TSH < 0.1 mIU/l.^36,40^ Subclinical hyperthyroidism is also associated with a higher heart rate, higher frequency of premature atrial, and ventricular beats and ventricular hypertrophy,^41,42^ although the latter is not seen in all studies.^43,44^ Interestingly, as seen in subclinical hypothyroidism, subclinical hyperthyroidism is also associated with increased carotid artery intima-media thickness.^45^ Regarding cardiac function, the possible association of subclinical hyperthyroidism with systolic and diastolic dysfunction is yet to be clarified, as there is evidence both for and against it.^41,46^

Overt hyperthyroidism is defined as low TSH with high free T4. It is associated with a hyperdynamic state, characterized by tachycardia, increased cardiac preload and contractility, and diminished systemic vascular resistance. In the short term, it may improve cardiovascular function, improving both systolic function and left ventricular relaxation. However, when sustained, it may induce high-output HF, even in the absence of underlying heart disease.^47^ Furthermore, overt hyperthyroidism is also strongly associated with atrial fibrillation.^48^ Overt hyperthyroidism has also been associated with pulmonary hypertension.^49^ Finally, untreated overt hyperthyroidism has consistently been associated with a higher risk of adverse cardiovascular events, as well as a higher risk of cardiovascular and all-cause mortality.^21,50^

## Modulation of diastolic function by thyroid hormones

Low thyroid hormone levels are associated with both systolic and diastolic dysfunction. However, both basic and clinical studies highlight that in hypothyroidism the diastolic abnormalities predominate.^51^ In a study of patients with subclinical hypothyroidism and matched controls, patients with subclinical hypothyroidism showed significant prolongation of the isovolumic relaxation time, increased A wave, and reduced E/A ratio (early to late ventricular filling velocities ratio).^27^ Furthermore, in a subgroup of patients that were reevaluated after thyroid hormone profile normalization, diastolic abnormalities were reversed and comparable with controls.^27^ Interestingly, the alterations in cardiac gene expression in HF is similar to the alterations observed in hypothyroidism.^52^

Thyroid hormones also enhance relaxation through improving bioenergetics. Treating subclinical hypothyroidism with levothyroxine improves cardiac phosphocreatine to ATP ratio,^53^ which may be related to the effects of thyroid hormones in cardiac mitochondrial function, including stimulation of cardiac mitochondrial biogenesis and improvement in oxidative phosphorylation. Moreover, vascular effects of thyroid hormones may contribute to enhance diastolic function as well.^3^ Experimental data also suggest that it may decrease myocardial stiffness as a rat model of propylthiouracil-induced hypothyroidism showed increased LV stiffness due to increased collagen deposition, despite overexpression of the larger and more compliant (N2BA) isoform of titin.^54^ Nevertheless, the effects on titin are not settled. Although thyroid hormones promote an increase in N2B/N2BA isoform ratio, it is possible that a higher titin phosphorylation mediated by PKG (secondary to improved endothelial function) and PKA (increased sensitivity to β-adrenergic stimulation) may outweigh the isoform shift effects on titin passive tension.

## Pathophysiology of HFpEF

HFpEF is a clinical syndrome consisting of symptoms and signs of HF that cannot be attributed to other causes, despite normal LV EF on echocardiographic evaluation. From a pathophysiological point of view, it is characterized by diastolic dysfunction with abnormal relaxation and/or increased passive stiffness that manifests as prolonged isometric relaxation, slow left ventricle filling and increased diastolic stiffness.^3,55^ The myocardial stiffening in HFpEF can be ascribed to the giant cytoskeletal protein titin at physiological sarcomere lengths or to the extracellular matrix at higher sarcomere lengths. HFpEF patients show both increased collagen content and titin-dependent stiffness, which is related to isoform shifts or decreased phosphorylation by PKA, PKG, and CAMKIIδ, though the latter seems to dominate.^56,57^ Changes in calcium kinetics, including increased diastolic calcium levels,^58^ are important contributors to abnormal relaxation in HFpEF. Impaired myocardial bioenergetics has also been proposed as a key mechanism for development of HFpEF, as it impairs an effective relaxation.^3^

Recently, the focus has shifted from cardiac mechanisms to extra-cardiac disturbances. Arterial stiffness, poor ventricular-arterial coupling, increased central volume, impaired vasodilation, pulmonary hypertension, endothelial dysfunction, and dysfunction of other tissues, including the lungs, skeletal muscle, adipose tissue, and kidneys, contribute to impaired cardiovascular reserve.^3,59^ Indeed, systemic involvement seems crucial in HFpEF. Patients are typically elderly, obese, with hypertension and diabetes, showing increased mortality due to non-cardiac causes when compared with HFrEF, and, therefore, warrant a strict control of the underlying comorbidities to improve cardiovascular reserve.

## Abnormal thyroid function in HF

Hypothyroidism is one of the most frequent endocrine abnormalities in the general population. A prevalence of 4–20% has been reported for the spectrum of hypothyroidism (subclinical or overt) in the general population.^1^ In HFpEF, the prevalence of hypothyroidism may be even higher as it is more common in women and the elderly – a group of individuals frequently diagnosed with HFpEF. In patients with HF (both HFrEF and HFpEF), non-thyroidal illness syndrome or low T3 syndrome is also common.^25^ Upregulation of D3 is one of the main mechanisms of low T3 levels in these patients. D3 overexpression is a common inflammatory response seen in non-thyroidal illness syndrome. Recent studies evidence that D3 expression is enhanced in certain pathological contexts in a cell-specific manner.^60^ Therefore, D3 upregulation in cardiomyocytes may contribute to the exacerbation of local cardiac hypothyroidism in association with decreased peripheral conversion of T4 to T3.^61^ This impaired peripheral conversion may be explained by the decreased activity of D2, seen in advanced heart disease.^62^ The exact mechanism by which D3 is enhanced is not fully understood; some studies show this may be mediated by inflammatory cytokines and catecholamines, both increased in HF.^63^ This cell-specific regulation is important to take into account because it may be masked due to the maintenance of constant circulating thyroid hormones concentration.^9^ Lower T3 levels have been associated with increased cardiovascular mortality in HF, in patients with cardiovascular disease, and in the general population.^25,64,65^ Low T3 levels have also been associated with higher in-hospital and 1-year mortality in patients hospitalized for acute decompensated HF.^66^ In a group of 89 consecutive patients with HFpEF, 22% had low T3 levels and 10% had elevated TSH. Low T3 was associated with markers of severity, including BNP and echocardiographic parameters of diastolic dysfunction.^67^ Changes in the gene expression associated with HF are similar to the fetal gene program and resembles that observed in hypothyroidism.^68^ Therefore, local cardiac hypothyroidism may reduce Ca^2+^ transients and induce an α-MHC to β-MHC shift.^68^ In an animal model of low T3 syndrome induced by chronic caloric deprivation, there was a significant decrease of SERCA2a and α-MHC with impairment of cardiac contraction and relaxation. T3 supplementation reverted these changes, highlighting the potential contribution of the low T3 syndrome to cardiac dysfunction.^69^

In patients with normal TSH, T3, and T4 serum levels – normal systemic thyroid function – important changes in thyroid hormone effects may still be present. Several animal studies suggest that HF is associated with local tissue hypothyroidism. Different animal models in recent years have shown that HFrEF and several important risk factors for HFpEF, including ischemia, hypertension, and diabetes mellitus, induce an increase in the expression of cardiac D3, and, consequently, a decrease in local cardiac T3 levels – locally impaired thyroid function.^70^ Most importantly, correction of cardiac hypothyroidism in animal models attenuated cardiac remodeling and myocardial dysfunction.^70^ As shown by Trivieri *et al.*, enhanced D2 activity in a rodent model increases cardiac T3 levels, improves cardiac inotropism and prevents deterioration of cardiac function after pressure overload.^71^ In addition, D2 upregulation also reverses the expression of genes associated with pathological remodeling.^71^

## Thyroid hormones as a therapeutic target in HFpEF

**Figure 1.**
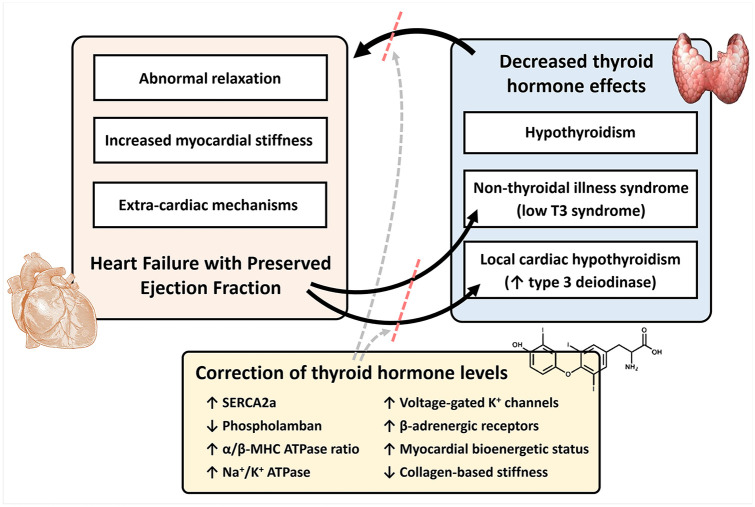
Decreased thyroid hormone effects worsen pathophysiologic changes of HFpEF. HFpEF is itself associated with low T3 syndrome and local cardiac hypothyroidism. Correction of tissue thyroid hormone levels has several effects that improve diastolic function and break the vicious cycle between cardiac dysfunction and decreased thyroid hormone effects, representing a promising therapeutic target in HFpEF. HFpEF, heart failure with preserved ejection fraction; MHC, myosin heavy chain; SERCA2a, sarco/endoplasmic reticulum calcium-ATPase; T3, triiodothyronine.

Given their cardiovascular effects, particularly concerning diastolic function, and the prognostic impact of thyroid function, modulation of thyroid hormone levels may constitute a promising therapeutic target in HFpEF (Figure 1). Indeed, diastolic dysfunction in hypothyroidism or subclinical hypothyroidism is reversible with thyroid hormone supplementation.^27^ A randomized clinical trial of patients with advanced HFrEF and low T3 levels showed improved neuroendocrine profile and ventricular performance after short-term intravenous T3.^72^ In an animal model of myocardial infarction-induced HF, T3 replacement to euthyroid levels improved both systolic and diastolic functions.^73^ Even without primary thyroid disease or abnormal hormone plasma levels, thyroid hormone supplementation may have beneficial effects. Correction of local tissue hypothyroidism with thyroid hormone supplementation improved diastolic function in animal models of HF.^74,75^ It is important to highlight that treatment with thyroid hormones may improve symptoms and morbidity in HFpEF, not only due to cardiac actions but also to extra-cardiac effects, including decreased adiposity and improved endothelial function, arterial compliance, and skeletal muscle function.^76^ Epicardial fat tissue has also been proposed as a cardiovascular risk factor, and it has been shown to be increased in hypothyroidism and in patients with HFpEF.^77^ Thus, the decrease of the epicardial fat tissue, and, possibly, the modulation of the profile of adipocytokines secreted by adipose tissue may contribute to the benefits of thyroid hormone supplementation.^77^

Thyroid hormone supplementation in HF has been studied mostly using HFrEF animal models.^51^ Furthermore, to this date, all clinical trials supplementing HF patients with thyroid hormones or their analogues refer to HFrEF (recently reviewed by Razvi *et al.*).^78^ Evidence from trials in HFrEF,^72^ and from trials in patients without HF, suggests a positive impact of thyroid hormone supplementation in diastolic function.^72,79,80^ However, clinical trials focused in HFpEF patients are necessary to fully understand the role of thyroid hormones as a potential therapeutic target for HFpEF.

The type of thyroid hormone to be used for the treatment of individuals with HF is an unsettled question. In patients with primary thyroid dysfunction, treatment with levothyroxine is the standard of care.^81^ The fact that patients with HF have decreased conversion of T4 into T3 suggests that a combination of levothyroxine and liothyronine could be associated with improvement of cardiac T3 levels. However, at the present time, there are no clinical studies to confirm this hypothesis. In patients with HF and low T3 syndrome, liothyronine may be the most appropriate approach from a pathophysiological perspective. Comparisons of liothyronine with levothyroxine or combined levothyroxine and liothyronine therapy in low T3 syndrome are also lacking.

The potential benefits of thyroid hormone supplementation should be weighed against the risks of overtreatment. Subclinical hyperthyroidism has been associated with myocardial hypertrophy and dysfunction, and increased risk of arrhythmias, mainly atrial fibrillation.^1^ It is also associated with increased risk of non-cardiovascular adverse consequences, including osteoporosis, anxiety, disturbances of sleep, and possibly cognitive dysfunction.^1^ Patients treated with thyroid hormones should be monitored regularly, and dosage must be adjusted according to plasma hormone levels to avoid overtreatment.

The minimization of potential adverse effects may be a key factor for successful use of thyroid hormones in HFpEF. A significant part of cardiovascular adverse effects from thyroid hormones supplementation is related to an increase in sympathetic activity. In order to minimize cardiovascular risk, an interesting approach may be the co-administration of a beta blocker. This would decrease the risk of arrhythmias, myocardial hypertrophy, and tachycardia-mediated myocardial dysfunction, without affecting the direct inotropic effects of thyroid hormones.^82^

An alternative approach to enhance thyroid hormone effects in patients with HFpEF, particularly in those with normal plasma thyroid hormones levels, would be the use of heart-specific TR agonists. This would avoid the possible extra-cardiac negative impact of thyroid hormone overtreatment, and would avoid the interference with the hypothalamus-pituitary-thyroid axis regulation. Although various thyromimetics that specifically target TRβ have been developed, no effective TRα-specific or heart-specific thyromimetic is known at this moment. DITPA (3,5-diodothyroproprionic acid) was also proposed as a potential thyromimetic with beneficial cardiac effects. DITPA has inotropic selectivity, without significant tachycardic effect.^83^ However, a multicenter clinical trial did not show improvement of clinical outcomes with DITPA in HFrEF.^83^

The modulation of the local cardiac deiodinase system is also an interesting target to increase the myocardial concentration of T3 without undesirable extra-cardiac effects. As stated earlier, recent evidence shows that D2 and D3 are expressed in a dynamic balance to control intracellular T3 levels and upregulation of D3 is involved in the genesis of a local cardiac hypothyroid state in HFpEF.^82,84,85^ Changes in redox balance may be central to the upregulation of D3. Reactive oxygen species (ROS) are known to disrupt peripheral deiodinase function, increasing D3 expression and activity, through mechanisms not yet fully understood.^86,87^ In addition, ROS production is also implicated in the pathophysiology of cardiac hypertrophy and remodeling, including in HFpEF.^84^ Thus, when redox imbalance is corrected, improvements in cardiac structure and function are expected. This was demonstrated in several studies using N-acetylcysteine, a precursor of glutathione, in different experimental models of HF.^88,89^ A significant part of these effects may be mediated by modulation of metabolism of thyroid hormones. Indeed, a recent study in a male rat model of myocardial infarction showed that N-acetylcysteine is able to revert the cardiac hypothyroid state and improve cardiac performance.^87^ Moreover, as N-acetylcysteine’s effects are not heart-specific, it may also interfere with deiodinase action, particularly D3, in other tissues, contributing to the prevention or resolution of the non-thyroidal illness syndrome.^87,90^

## Conclusion

Thyroid hormones have an important role in cardiac and vascular function through genomic and non-genomic mechanisms. HFpEF is a clinical syndrome characterized by diastolic dysfunction and extra-cardiac disturbances, for which there is no proven therapy to improve its prognosis. Thyroid hormone axis modulation holds potential for improving the prognosis in patients with HFpEF. Although different therapeutic approaches may allow the optimization of thyroid hormone effects in HFpEF, it is still not clear which have more potential for clinical use. Furthermore, a more comprehensive characterization of the thyroid system in HFpEF patient cohorts and further pre-clinical tests in animal models of HFpEF are needed to hasten translation to clinical trials in a disease that has so far eluded conventional therapeutic approaches.
